# Trimethylamine N-Oxide Was Not Associated With 30-Day Left Ventricular Systolic Dysfunction in Patients With a First Anterior ST-Segment Elevation Myocardial Infarction After Primary Revascularization: A Sub-analysis From an Optical Coherence Tomography Registry

**DOI:** 10.3389/fcvm.2020.613684

**Published:** 2020-12-23

**Authors:** Jinying Zhou, Shiqin Yu, Yu Tan, Peng Zhou, Chen Liu, Zhaoxue Sheng, Jiannan Li, Runzhen Chen, Shihua Zhao, Hongbing Yan

**Affiliations:** ^1^Department of Coronary Heart Disease, Fuwai Hospital, State Key Laboratory of Cardiovascular Disease, National Centre for Cardiovascular Diseases, Chinese Academy of Medical Sciences and Peking Union Medical College, Beijing, China; ^2^Magnetic Resonance Centre, Fuwai Hospital, State Key Laboratory of Cardiovascular Disease, National Centre for Cardiovascular Disease, Chinese Academy of Medical Sciences and Peking Union Medical College, Beijing, China; ^3^Xiamen Cardiovascular Hospital, Xiamen University, Fujian, China; ^4^Fuwai Hospital, Chinese Academy of Medical Sciences, Shenzhen, China

**Keywords:** trimethylamine N-oxide, ST-segment elevation myocardial infarction, left ventricular systolic dysfunction, gut metabolite, cardiovascular magnetic resonance

## Abstract

**Objective:** Left ventricular systolic dysfunction (LVSD) after ST-segment elevation myocardial infarction (STEMI) is associated with poor outcome. Trimethylamine N-oxide (TMAO), a gut metabolite, is linked to cardiovascular diseases but its relationship with LVSD after STEMI remains unclear. The present study therefore aimed to investigate the relationship between TMAO and LVSD at 30 days after a first anterior STEMI.

**Methods:** This was a sub-study from the OCTAMI (Optical Coherence Tomography Examination in Acute Myocardial Infarction) registry. Eligible patients were included in current study if they: (1) presented with a first anterior STEMI; (2) had available baseline TMAO concentration; (3) completed a cardiovascular magnetic resonance examination at 30 days after STEMI. LVSD was defined as left ventricular ejection fraction < 50%. Associations between TMAO and left ventricular ejection fraction, infarct size and left ventricular global strain were examined.

**Results:** In total, 78 patients were included in final analysis. Overall, TMAO was moderately associated with peak cTnI (*r* = 0.27, *p* = 0.01), age (*r* = 0.34, *p* < 0.01), and estimated glomerular filtration rate (*r* = −0.30, *p* < 0.01). At 30-day follow-up, 41 patients were in the LVSD group and 37 in the non-LVSD group. Baseline TMAO levels were not significantly different between the two groups (LVSD vs. non-LVSD: median 1.9 μM, 25−75th percentiles 1.5–3.3 μM vs. median 1.9 μM, 25−75th percentiles 1.5–2.7 μM; *p* = 0.46). Linear regression analyses showed that TMAO was not associated with left ventricular ejection fraction, infarct size or left ventricular global strain at 30 days (all *p* > 0.05).

**Conclusions:** TMAO was not significantly correlated with 30-day LVSD in patients with a first anterior STEMI after primary revascularization.

**Clinical Trial Registration:** www.ClinicalTrials.gov, identifier: NCT03593928.

## Introduction

Left ventricular systolic dysfunction (LVSD) and heart failure are frequently observed after ST-segment elevation myocardial infarction (STEMI) (1–3) and patients with left anterior descending artery as the culprit vessel face even higher risks (4). Thus, it's important to optimize risk stratification after STEMI and find out novel treatment targets for preventing heart failure.

Trimethylamine N-oxide (TMAO), a gut-derived metabolite (5), has been related to long-term cardiovascular risks in various populations (6–14), including those with established chronic heart failure (7, 12, 14) or acute heart failure (9). However, TMAO was unable to predict risks of death and recurrent myocardial infarction at 6 months after acute myocardial infarction (11) or to predict cardiovascular risks in patients with type 1 diabetes mellitus (15), high risk type 2 diabetes mellitus (16), or previous myocardial infarction (17). It was also reported that TMAO was associated with advanced left ventricular diastolic dysfunction, but not with systolic dysfunction in patients with chronic systolic heart failure (8). Results from animal studies remain controversial. Organ and her colleagues reported that TMAO exacerbated pulmonary edema, cardiac enlargement and left ventricular ejection fraction (LVEF) decline in pressure-overloaded mice heart failure model (18). Further, they showed that removing dietary TMAO had a beneficial effect on the myocardium in another murine heart failure model (19). In contrast, a four- to five-fold increased plasma TMAO alleviated diastolic dysfunction in pressure-overloaded rat heart failure (20). Nevertheless, there was a negative effect of TMAO on aortic atherosclerotic lesions in ApoE knockout mice (21). Evidence to date supports detrimental effects of TMAO that include promoting inflammation (22–24), impairing cardiomyocyte (25), disrupting energy metabolism and increasing oxidative stress (26), which may play a role in heart failure after myocardial infarction.

Cardiovascular magnetic resonance (CMR) has become an intriguing imaging modality by providing a comprehensive and multifaceted evaluation of the heart (27). LVEF is widely used in clinical practice and CMR provides more accurate measurements compared to echocardiography (28). CMR allows the unique detection of infarcted size or fibrosis by late gadolinium enhancement (LGE) (29) and features of injured myocardium such as microvascular obstruction (MVO) and intramyocardial hemorrhage (30). Left ventricular strain by CMR feature tracking analysis has emerged in the past decade as a useful tool for quantitative evaluation of deformation (31) and provides additional prognostic values following myocardial infarction (32).

Therefore, we aimed to investigate the possible association between baseline TMAO and consequential development of LVSD at 30 days, as assessed by CMR, in patients after a first anterior STEMI and primary percutaneous coronary intervention.

## Methods

### Study Population

**Figure 1:**
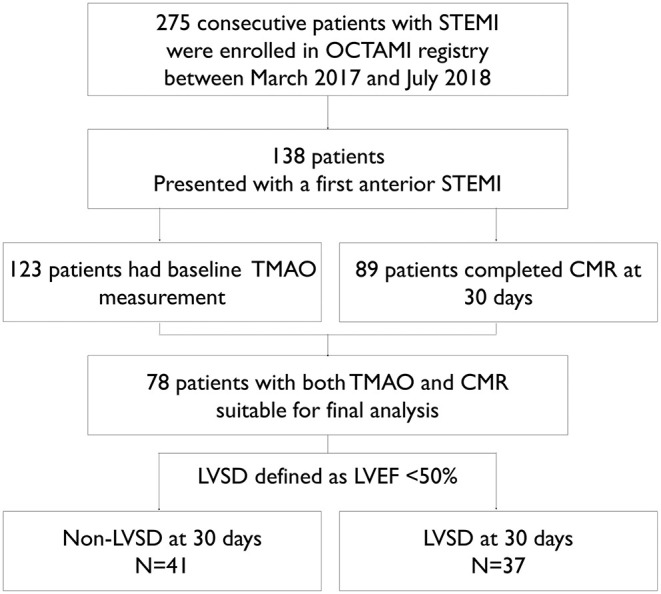
Study flow. CMR indicates cardiovascular magnetic resonance; LVEF, left ventricular ejection fraction; LVSD, left ventricular systolic dysfunction; OCTAMI registry, Optical Coherence Tomography Examination in Acute Myocardial Infarction registry (NCT03593928); STEMI, ST-segment elevation myocardial infarction; TMAO, trimethylamine-N-oxide.

Data were used from the OCTAMI (Optical Coherence Tomography Examination in Acute Myocardial Infarction) registry (NCT03593928), which enrolled a prospective, consecutive, observational, single-center cohort of patients with STEMI for optical coherence tomography examination at the culprit lesion (33–35). Study flow is shown in Figure 1. We approached all patients from the registry who presented with a first anterior STEMI and had available baseline TMAO level and 30-day CMR follow-up between March 2017 and July 2018. STEMI was defined as persistent symptoms suggesting myocardial ischemia, elevated troponin I levels, and ST-segment elevation in at least 2 contiguous leads or new left bundle branch block on the 18-lead electrocardiogram (36). Baseline LVEF was measured by echocardiography as part of standard routine and results were extracted from electronic medical records. Left ventricular evaluation by CMR was scheduled at 30 days after the index STEMI. This study complied with the principles of the Declaration of Helsinki and was approved by the review board at Fuwai Hospital. All patients provided written informed consent.

### Procedural Data

Patients received initial oral treatment of 300 mg aspirin, 180 mg ticagrelor or 600 mg clopidogrel, and intravascular infusions of 75–100 IU/kg heparin prior to coronary angiography. Percutaneous coronary intervention was performed via radial or femoral access. Infusions of glycoprotein IIb/IIIa receptor inhibitors were at the discretion of the operators. The culprit vessel was determined primarily by coronary angiography and corroborated with electrocardiogram and echocardiography.

### Biomarker Analysis

Peripheral blood samples were collected before heparinization using vacutainer tubes containing ethylenediamine tetra-acetic acid, maintained at 4°C and processed within 3 h. Then, plasma samples were stored at −80°C and thawed once for analysis. Plasma levels of TMAO were quantified by stable isotope dilution high performance liquid chromatography with online tandem electrospray ionization mass spectrometry using an API 3200 triple quadrupole mass spectrometer (AB SCIEX, Framingham, MA) with a d9-(trimethyl)-labeled internal standard as described previously (37). In addition, cardiac troponin I (cTnI) and NT-proBNP were measured as routine biochemical work-up during the hospital stay. As a standard care, cTnI was measured at least 3 times within 24 h after admission and daily until normalization and then every 2 days before discharge; NT-proBNP was measured on admission, immediately after and on the second day after the index procedure and then up to physicians. Results of cTnI and NT-proBNP during hospitalization were retrieved from electronic medical records and baseline values and peak values were reported.

### CMR Image Acquisition

CMR imaging was performed on a 3.0-Tesla scanner (Discovery MR750, GE Healthcare, Milwaukee, USA) with a phased-array cardiovascular coil, using electrocardiographic and respiratory gating. Cine images were acquired in three long-axis views (two-chamber, four-chamber, and left ventricular outflow tract) and short-axis views encompassing the entire left ventricle, using balanced steady state free precession sequence. Acquisition parameters were as follows: 3.3/1.7 ms repetition time/echo time; 320*320 mm field of view; 224*192 matrix; 50° flip angle; 46–60 ms temporal resolution; 8 mm slice thickness; 2 mm slice gap. LGE images were acquired 10 to 15 min after intravenous administration of gadolinium-DTPA (Magnevist, Bayer, Berlin, Germany) at a dose of 0.2 mmol/kg, using a segmented phase-sensitive inversion recovery Turbo Fast Low Angle Shot sequence at the same views as cine images in end diastole. Typical imaging parameters were: 360*360 mm field of view; 224*192 matrix, 6.0/2.8 ms TR/TE time; 25° flip angle; 8 mm slice thickness; 2 mm slice gap; 300 ms TI.

### CMR Image Analysis

All CMR images were analyzed with CVI42 (Circle Cardiovascular Imaging Inc., Calgary, Canada) by radiologists with more than 5-year experience of CMR imaging. Infarct size was quantified by LGE detected as +5 SDs over the signal intensity of normal myocardium and the percentage of enhanced myocardium mass of left ventricular mass was recorded. MVO was identified as areas of hypointensity within the region of hyperenhanced infarct tissue. Both the presence of MVO and quantitative measurements as percentage of left ventricular mass were reported. Feature tracking analysis provided peak left ventricular global strain parameters, including: global radial strain (GRS), global circumferential strain (GCS) and global longitudinal strain (GLS). Briefly, endocardial and epicardial contours of the left ventricle throughout the cardiac cycle were automatically tracked by contours that were manually drawn at end diastole. Papillary muscles were assigned to the left ventricular volume. Other cardiac parameters were computed automatically based on manually traced endocardial and epicardial contours of left ventricular myocardium on short-axis cine at end diastole and end systole, respectively. Inter-observer and intra-observer variability analyses of CMR strain were determined from 10 randomly selected STEMI patients (data not shown).

### Statistical Analysis

Continuous variables were presented as means with standard deviations (SD) or medians with 25th and 75th percentiles and were compared between two groups with *t* test or Mann Whitney test. In addition, Kruskal–Wallis test was applied to comparing continuous variables among four groups. Categorical variables were presented as counts and percentages and were compared with chi-square test. LVSD was defined as LVEF <50%. Bivariate correlations were evaluated with Pearson's correlation. Univariate and multivariate linear regression analyses were performed to detect possible associations between TMAO and left ventricular parameters (LVEF, infarct size, MVO, GCS, GRS, GLS) at 30 days. Model 1 included adjustments for age and estimated glomerular filtration rate (eGFR), and Model 2 included adjustments for age, eGFR, peak cTnI and peak NT-proBNP. A 2-tailed *p* < 0.05 was considered statistically significant. All statistical analyses were performed using SPSS software, version 24 (IBM, Armonk, NY).

## Results

### Baseline Characteristics

From March 2017 to July 2018, 275 patients were enrolled in the OCTAMI registry and 138 of them presented with a first anterior STEMI. Finally, seventy-eight patients with both baseline TMAO level and 30-day CMR follow-up were qualified for the current study. Comparisons of baseline characteristics between included and excluded groups of patients with a first anterior STEMI were reported in Supplementary Table 1. Among the seventy-eight patients, the median TMAO level was 1.9 μM with 25th percentile of 1.5 μM and 75th percentile of 2.9 μM. According to LVEF at 30 days, 41 patients were in the LVSD group and 37 in the non-LVSD group. Baseline characteristics between groups are shown in Table 1. There was no significant difference of TMAO levels between the two groups. Baseline LVEF was similar between the two groups. However, the LVSD group had higher peak NT-proBNP, and higher peak cTnI. Moreover, there was a significantly higher percentage of females in the group of LVSD. No significant differences between groups were observed regarding age, body mass index, medical history, procedural data, and medications at discharge.

**Table 1:** Baseline characteristics.

	**LVSD at 30 days *N* = 41**	**Non-LVSD at 30 days *N* = 37**	***p* value**
TMAO, μM	1.9 (1.5, 3.3)	1.9 (1.5, 2.7)	0.46
**Demographics**			
Age, years	54.7 ± 10.6	54.2 ± 8.9	0.83
Male	29 (70.7)	35 (94.6)	**0.01**
BMI, kg/m^2^	25.7 ± 3.9	25.8 ± 2.9	0.85
**Medical history**			
Smoker	20 (48.8)	25 (67.6)	0.09
Hypertension	17 (41.5)	16 (43.2)	0.87
Hyperlipidaemia	32 (78.0)	30 (81.1)	0.74
Diabetes	11 (26.8)	8 (21.6)	0.59
Stroke	5 (12.2)	1 (2.7)	0.13
PAD	1 (2.4)	0 (0)	0.53
**Baseline LV parameters**			
LVEDD, mm	49.8 ± 4.6	49.8 ± 4.1	0.95
LVEF, %	50.4 ± 6.9	53.1 ± 6.0	0.07
**Procedural data**			
MVD	25 (61.0)	25 (67.6)	0.54
Initial TIMI flow 0–1	33 (80.5)	24 (64.9)	0.12
Aspiration	24 (58.5)	24 (64.90)	0.57
PTCA	1 (2.4)	3 (8.1)	0.27
Stenting	38 (92.7)	33 (89.2)	0.59
Total length, mm	30.6 ± 10.9	28.8 ± 12.8	0.53
Minimal diameter, mm	3.0 ± 0.4	3.0 ± 0.5	0.81
Final TIMI flow 3	40 (97.6)	37 (100)	0.53
**Laboratory indexes**			
Baseline NT-proBNP, ng/mL	70.1 (34.9, 243.4)	90.1 (20.6, 300.9)	0.19
Peak NT-proBNP, ng/mL	2,258.8 (1,141.4, 3,914.9)	1,285.9 (660.5, 2,114.2)	**0.01**
Baseline cTnI, ng/mL	0.5 (0.1, 1.7)	0.2 (0.0, 1.4)	0.30
Peak cTnI, ng/mL	52.8 (21.3, 91.2)	21.0 (7.4, 36.6)	**<0.01**
Hs-CRP, mg/L	5.6 ± 4.3	5.2 ± 3.9	0.66
Hemoglobin, g/L	150.8 ± 18.0	152.9 ± 14.7	0.59
eGFR, ml/min/1.732m^2^^*^	96.8 ± 20.6	101.6 ± 22.5	0.33
HbA1c, %	6.4 ± 1.7	6.3 ± 1.2	0.36
LDL-C, mmol/L	119.5 ± 32.2	113.1 ± 29.8	0.37
**Medications at discharge**			
Aspirin	41 (100)	37 (100)	–
P2Y12	41 (100)	37 (100)	–
Ticagrelor	27 (65.9)	20 (54.1)	0.29
Clopidogrel	14 (34.1)	17 (45.9)	0.29
ACEIs/ARBs	35 (85.4)	34 (91.9)	0.30
Beta blockers	40 (97.6)	34 (91.9)	0.26
Diuretics	17 (41.5)	9 (24.3)	0.11
Statin	38 (92.7)	35 (94.6)	0.73

*Continuous variables are presented as mean ± SD or medians (25−75th percentiles), and categorical variables are reported as counts (%). All p values <0.05 are in bold. ACEIs/ARBs indicates angiotensin-converting enzyme inhibitors/angiotensin receptor blockers; BMI, body mass index; cTnI, cardiac troponin I; Hs-CRP, high-sensitivity C reactive protein; LDL-C, low-density lipoprotein cholesterol; LVEF, left ventricular ejection fraction; LVEDD, left ventricular end-diastole diameter; LVSD, left ventricular systolic dysfunction; MVD, multi-vessel disease; NT-proBNP, N-terminal pro B-type Natriuretic Peptide; PAD, peripheral artery disease; TMAO, trimethylamine-N-oxide*.

* *Estimated glomerular filtration rate (eGFR) was calculated according to the Modification of Diet in Renal Disease formula*.

### Associations of TMAO With Traditional Biomarkers

**Figure 2:**
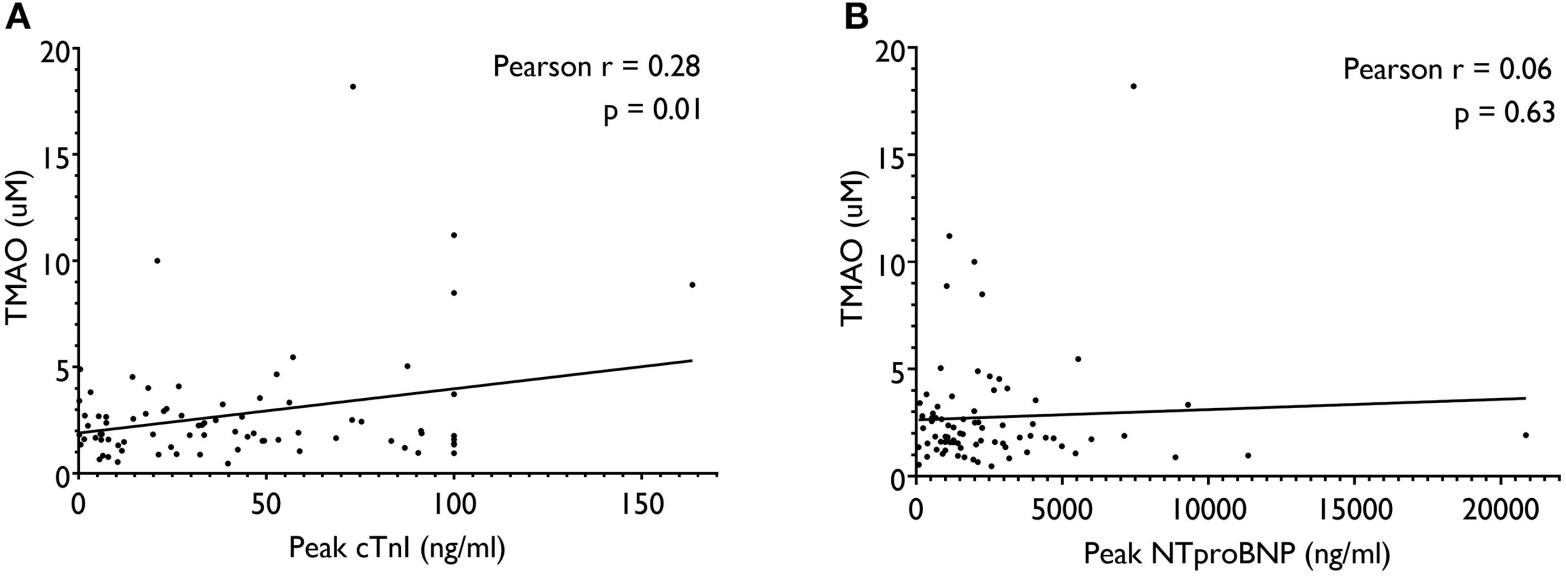
Pearson correlations between TMAO and traditional biomarkers, including peak cTnI **(A)** and peak NT-proBNP **(B)**. cTnI indicates cardiac troponin I; NT-proBNP, N-terminal pro B-type Natriuretic Peptide; TMAO, trimethylamine-N-oxide.

Bivariate relationships between TMAO and traditional biomarkers are presented in Figure 2. TMAO was not associated with peak NT-proBNP (*r* = 0.06, *p* = 0.63), but had a statistically significant and moderate association with peak cTnI (*r* = 0.32, *p* = 0.01). Besides, TMAO was also significantly correlated with age (*r* = 0.34, *p* < 0.01) and eGFR (*r* = −0.30, *p* = 0.01) in the current study cohort.

### CMR Parameters at 30-Day Follow-Up

Between-group levels of left ventricular CMR parameters are shown in Table 2. The LVSD group, compared with the non-LVSD group, presented significantly larger end-systolic volume, larger end-diastole volume, and larger infarct size. We observed no significant difference of MVO presence or MVO percentages between the two groups. In accordance with traditional parameters, left ventricular feature tracking strain analyses showed that GRS, GCS, and GLS were significantly worse in patients with LVSD than in those without LVSD at 30 days. In bivariate correlation analysis, GRS, GCS, and GLS were significantly associated with peak cTnI and peak NT-proBNP but not with TMAO (Table 3).

**Table 2:** Left ventricular CMR parameters at 30-day follow-up.

	**LVSD at 30 days *N* = 41**	**Non-LVSD at 30 days*N* = 37**	***p* value**
**Traditional parameters**			
LVEF, %	39.7 ± 7.1	57.3 ± 4.9	<0.01
EDV, ml	154.6 ± 34.2	136.1 ± 33.5	0.02
ESV, ml	93.8 ± 25.7	59.1 ± 18.4	<0.01
SV, ml	60.9 ± 16.3	77.5 ± 18.2	<0.01
HR, bpm	68.6 ± 11.5	61.4 ± 11.5	<0.01
CO, L	4.14 ± 1.23	4.74 ± 1.13	0.03
Infarct size, % LV mass	20.6 ± 10.0	7.4 ± 6.1	<0.01
MVO presence	22 (53.7)	18 (48.6)	0.66
MVO, %LV mass	0.7 ± 0.9	0.6 ± 0.8	0.72
**Left ventricular strain**			
GRS, %	17.4 ± 4.1	26.4 ± 5.2	<0.01
GCS, %	−11.7 ± 2.6	−16.2 ± 2.1	<0.01
GLS, %	−9.9 ± 2.3	−12.4 ± 2.2	<0.01

*Continuous variables are presented as mean ± SD. CMR indicates cardiovascular magnetic resonance imaging; CO, cardiac output; ESV, end-systole volume; EDV, end-diastole volume; GCS, global circumferential strain; GLS, global longitudinal strain; GRS, global radial strain; HR, heart rate; LVSD, left ventricular systolic dysfunction; LVEF, left ventricular ejection fraction; SV, stroke volume*.

**Table 3:** Pearson's correlation coefficients between biomarkers and CMR parameters.

	**LVEF**	**EDV**	**ESV**	**SV**	**HR**	**CO**	**Infarct size**	**MVO**	**GRS**	**GCS**	**GLS**
TMAO, μM	−0.13	−0.10	−0.01	−0.17	−0.02	−0.18	−0.05	0.01	−0.17	0.18	0.07
Peak NT-proBNP, pg/mL	−0.29^*^	−0.02	0.14	−0.24^*^	0.15	−0.16	0.34^**^	−0.18	−0.25^*^	0.27*	0.30^**^
Peak cTnI, ng/mL	−0.44^**^	0.17	0.34^**^	−0.19	0.21	−0.05	0.48^**^	−0.11	−0.39^**^	0.41^**^	0.31^**^

* p < 0.05;

** *p < 0.01. Abbreviations as in Tables 1, 2*.

### Relationship Between TMAO and LVSD

**Figure 3:**
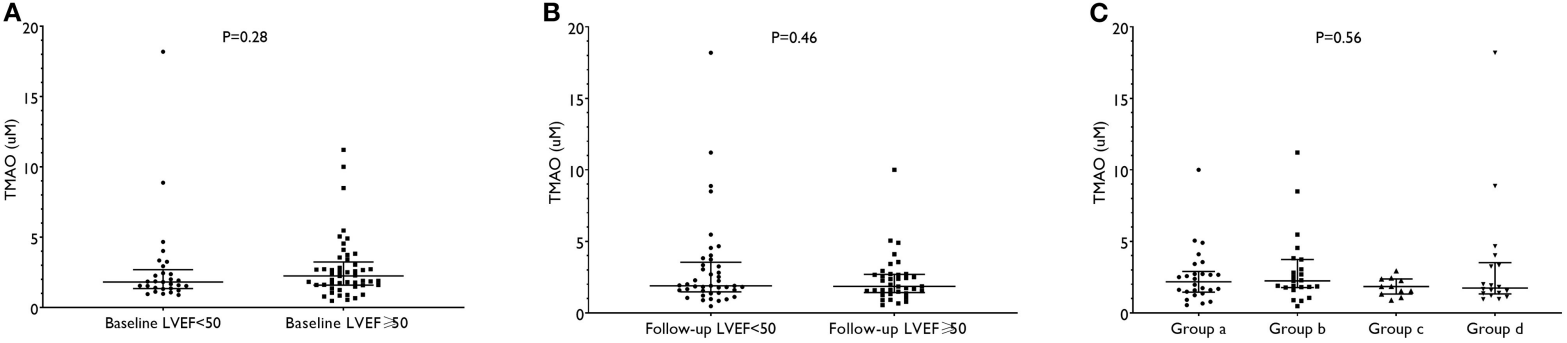
TMAO levels grouped by LVSD at baseline **(A)**, at 30-day follow-up **(B)** and both **(C)**. In **(C)**, groups are defined as follows: Group a includes patients not presenting LVSD at both baseline and 30 days; Group b includes patients presenting with LVSD solely at 30 days; Group c includes patients presenting with LVSD solely at baseline; Group d includes patients presenting persistent LVSD at both baseline and 30-days. LVEF indicates left ventricular ejection fraction; LVSD, left ventricular systolic dysfunction; TMAO, trimethylamine-N-oxide.

Baseline TMAO levels between patients with and without LVSD at 30 days were not significantly different (Figure 3B). Then, we re-grouped patients according to LVEF at baseline and we observed no significant differences of TMAO levels between groups (Figure 3A). We further grouped patients according to the change of LVEF from baseline to 30 days. As a result, twenty-six patients had no LVSD at baseline or at 30 days (Group a); twenty-three patients had LVSD solely at 30 days (Group b); eleven patients had LVSD solely at baseline (Group c); and eighteen patients presented persistent LVSD at both baseline and at 30 days (Group d). Nevertheless, there were no significant differences of TMAO levels among the four groups (Figure 3C). In linear regression analyses (Table 4), TMAO was not significantly correlated with LVEF, infarct size, MVO, GRS, GCS, or GLS at 30 days.

**Table 4:** Linear regression analyses of associations between TMAO and left ventricular parameters at 30 days.

	**Unadjusted**		Model 1^*^		Model 2^**^	
	**β (95% CI)**	***p* value**	**β (95% CI)**	***p* value**	**β (95%CI)**	***p* value**
**For LVEF**						
TMAO, μM	−0.52 (−1.43, 0.39)	0.26	−0.40 (−1.41, 0.60)	0.43	−0.01 (−0.96, 0.95)	0.99
**For infarct size**						
TMAO, μM	−0.18 (−1.10, 0.72)	0.68	−0.36 (−1.36, 0.65)	0.48	−0.80 (−1.68, 0.09)	0.08
**For MVO**						
TMAO, μM	0.01^¶^ (−0.07, 0.08)	0.96	0.01^§^ (−0.10, 0.06)	0.61	−0.03 (−0.11, 0.06)	0.51
**For GRS**						
TMAO, μM	0.21 (−0.06, 0.48)	0.12	0.15 (−0.14, 0.45)	0.31	0.06 (−0.23, 0.34)	0.69
**For GCS**						
TMAO, μM	−0.43 (−0.97, 0.12)	0.12	−0.33 (−0.93, 0.27)	0.28	−0.14 (−0.74, 0.45)	0.63
**For GLS**						
TMAO, μM	0.07 (−0.15, 0.29)	0.54	0.09 (−0.16, 0.33)	0.49	0.06 (−0.18, 0.29)	0.65

* Model 1: adjusted for age and eGFR;

** Model 2: adjusted for age, eGFR, peak NTpro-BNP and peak cTnI;

¶ β with three decimals was 0.002;

§ *β with three decimals was −0.002. Abbreviations as in Tables 1, 2*.

## Discussion

The present study showed that TMAO levels were not significantly different between patients with or without LVSD at 30 days after a first anterior STEMI. Although TMAO was significantly and moderately associated with peak cTnI, it had no statistically significant association with LVEF, infarct size, MVO or left ventricular global strain at 30 days in the current study cohort.

TMAO is a metabolite derived from gut flora (5) and is related to cardiovascular diseases (6–11, 14). However, its prognostic utility remains controversial, due to different follow-up intervals (11), study populations (16, 17, 38), geographical regions (10, 39), and ethnicities (40, 41). In addition, it has been reported that the core intestinal microbiota in patients with heart failure were usually altered (42), resulting in significantly increased circulating TMAO levels (43). It is intriguing that Tang et al. reported that in chronic heart failure, TMAO was associated with advanced left ventricular diastolic dysfunction, but not systolic dysfunction or inflammatory biomarkers (8). Consistently, the present study showed no significant relationship between baseline TMAO level and 30-day LVSD in patients with a first anterior STEMI. To note, the average level of TMAO in current study was relatively lower compared with other studies except for that reported by Liu et al. (44), which might result from ethnic differences and regional diet characteristics (45). Moreover, the predictive value of TMAO for cardiovascular events was concentration-dependent, and probably with a threshold-type association, as Salzano et al. reported that TMAO as a continuous variate was not associated with a composite outcome of mortality and hospitalization for heart failure but TMAO level exceeding a cut-off of 5 mmol/L was significantly associated with fourfold increased event rates (46). Similarly, another study (10) included two independent large cohort, the Cleveland Cohort and the Swiss Cohort, and results showed that patients in the third quartile of TMAO level in the former cohort but not those in the latter cohort presented significant higher cardiovascular risks, which might be due to a higher TMAO level in the former cohort than in the latter one (the third quartile in the former cohort: 4.28–7.89 uM; the third quartile in the latter cohort: 2.87–4.84 uM). Although previous studies reported TMAO as a prognostic biomarker in patients with heart failure, only a few studies (15–17, 38) evaluated TMAO in patients without established heart failure and surprisingly, these studies all reported non-significant prognostic value of TMAO for cardiovascular outcomes after adjustments for traditional parameters. To our knowledge, our study is the first to investigate the role of TMAO in LVSD after a first anterior STEMI and we did not observe a significant relationship between the two. Further, a recent bidirectional mendelian randomization analysis between TMAO and cardiometabolic diseases supported that type 2 diabetes mellitus and chronic kidney diseases increased TMAO and that observational evidence for TMAO's role in cardiovascular diseases probably resulted from confounders or reverse casualty (47).

TMAO is also linked to the pathogenesis of atherosclerosis by promoting macrophage cholesterol accumulation and altering sterol metabolism in mice models (48). However, we did not observe correlations between TMAO and baseline cholesterol levels. Besides, we observed significant correlations between TMAO level with age, eGFR and peak cTnI. As we previously reported, higher TMAO level was related with higher prevalence of plaque rupture (34) and heavier atherosclerotic burden (33), which were reported to increase the risk of impaired myocardial reflow (49, 50). On the other hand, there was no significant association between peak NT-proBNP and TMAO in the present study. However, Tang et al. reported a significant correlation between TMAO and B-type natriuretic peptide measured from fasting blood samples in chronic heart failure patients (7). This difference might result from different disease scenarios and distinct sampling times.

Several limitations warrant mention. First, this was not a pre-specified sub-analysis and results should be considered as hypothesis generating and be interpreted with caution. Second, we enrolled a limited number of patients in the current study. However, all patients were from a continuously screened and prospectively collected STEMI cohort, which could minimize selection bias. Third, CMR was limited to 30-day follow-up, and not performed during hospitalization. Thus, we collected baseline LVEF from echocardiography for grouping patients but not in further correlation analysis or regression analysis. It was also reported that in patients with anterior STEMI, LVEF measurements from echocardiography and CMR were within acceptable limits of agreement (51). Finally, the intrinsic nature of observational studies was insufficient to draw or deny a causal relationship between TMAO and short term LVSD. Future studies with larger sample size and possible interventions on TMAO levels are needed.

## Conclusion

In conclusion, baseline TMAO levels were not significantly associated with LVEF, infarct size, or left ventricular global strain at 30-day follow-up in patients with first ever anterior STEMI after primary percutaneous coronary interventions. Although TMAO was moderately correlated with peak cTnI, it was not associated with left ventricular systolic dysfunction at 30 days follow-up.

## Data Availability Statement

The original contributions presented in the study are included in the article/Supplementary Materials, further inquiries can be directed to the corresponding author/s.

## Ethics Statement

The studies involving human participants were reviewed and approved by the Review Board of Fuwai Hospital. The patients/participants provided their written informed consent to participate in this study.

## Author Contributions

JZ analyzed and interpreted the complete data, and was a major contributor in writing the manuscript. SY analyzed and interpreted the patient data regarding cardiovascular magnetic resonance imaging results, and was a major contributor in writing the manuscript. YT contributed to the analysis of baseline TMAO concentration. PZ and CL played a leading role in patient enrolment and conducting the registry study. ZS, JL, and RC collected and analyzed the patient data regarding clinical characteristics. HY and SZ supervised the study and were responsible in funding support. All authors contributed to the article and approved the submitted version.

## Conflict of Interest

The authors declare that the research was conducted in the absence of any commercial or financial relationships that could be construed as a potential conflict of interest.
