# Supplementary material for: Trimethylamine N-Oxide Was Not Associated With 30-Day Left Ventricular Systolic Dysfunction in Patients With a First Anterior ST-Segment Elevation Myocardial Infarction After Primary Revascularization: A Sub-analysis From an Optical Coherence Tomography Registry
Source: Front Cardiovasc Med. 2020 Dec 23;7:613684. doi: 10.3389/fcvm.2020.613684 (PMC7786017; doi:10.3389/fcvm.2020.613684)
Supplement: Supplementary file 1 [file Table_1.DOCX]

Supplementary Material

# Supplementary Table

## Supplementary Table 1 Baseline characteristics between excluded and included groups of patients with a first anterior ST-segment elevation myocardial infarction

|  | Excluded  N=60 | Included  N=78 | *p* values |
| --- | --- | --- | --- |
| Demographics |  |  |  |
| Age, years | 58.0±11.8 | 54.4±9.74 | 0.06 |
| Male, n (%) | 43(71.7%) | 64(82.1%) | 0.21 |
| BMI, kg/m^2^ | 26.1±3.12 | 25.7±3.43 | 0.46 |
| Medical history |  |  |  |
| Smoker, n (%) | 39(65.0%) | 45(57.7%) | 0.49 |
| Hypertension, n (%) | 40(66.7%) | 33(42.3%) | **0.01** |
| Hyperlipidaemia, n (%) | 48(80.0%) | 62(79.5%) | 1.00 |
| Diabetes, n (%) | 19(31.7%) | 19(24.4%) | 0.45 |
| Stroke, n (%) | 3(5.00%) | 6(7.69%) | 0.73 |
| PAD, n (%) | 0(0.00%) | 1(1.28%) | 1.00 |
| Baseline LV parameters |  |  |  |
| LVEDD, mm | 50.0±4.25 | 49.8±4.35 | 0.82 |
| LVEF, % | 53.9±6.10 | 51.7±6.64 | **0.05** |
| Procedural data |  |  |  |
| MVD | 38(63.3%) | 50(64.1%) | 1.00 |
| Initial TIMI flow 0-1 | 44(73.3%) | 57(73.1%) | 1.00 |
| Aspiration | 35(58.3%) | 48(61.5%) | 0.84 |
| PTCA |  |  | 0.70 |
| Stenting | 58(96.7%) | 71(91.0%) | 0.30 |
| Total length | 32.7±14.7 | 29.8±11.8 | 0.23 |
| Minimal diameter | 3.08±0.40 | 3.04±0.39 | 0.55 |
| Final TIMI flow 3 | 60(100%) | 77(98.7%) | 1.00 |
| Laboratory indexes |  |  |  |
| Baseline NT-proBNP, ng/mL | 107(35.9,739) | 76.8(27.0,287) | 0.11 |
| Peak NT-proBNP, ng/mL | 1630(598,3057) | 1637(881,3038) | 0.46 |
| Baseline cTnI, ng/mL | 0.57(0.08,3.49) | 0.23(0.04,1.58) | 0.19 |
| Peak cTnI, ng/mL | 23.8(12.9,47.4) | 32.6(10.5,58.8) | 0.27 |
| Hs-CRP, mg/L | 6.09±4.04 | 5.45±4.12 | 0.36 |
| Haemoglobin, g/L | 147±18.0 | 152±16.5 | 0.11 |
| eGFR, ml/min/1.732m^2^* | 97.4±22.9 | 99.1±21.5 | 0.67 |
| HbA1c, % | 6.64±1.62 | 6.36±1.47 | 0.30 |
| LDL-C, mmol/L | 2.96±0.96 | 3.01±0.80 | 0.77 |
| Medications at discharge |  |  |  |
| Aspirin, n (%) | 60(100%) | 78(100%) | . |
| P2Y12, n (%) | 60(100%) | 78(100%) | . |
| Ticagrelor, n (%) | 34(56.7%) | 47(60.3%) | 0.80 |
| Clopidogrel, n (%) | 26(43.3%) | 31(39.7%) | 0.80 |
| ACEIs/ARBs, n (%) | 47(78.3%) | 69(88.5%) | 0.17 |
| Beta blockers, n (%) | 60(100%) | 74(94.9%) | 0.13 |
| Diuretics, n (%) | 18(30.0%) | 26(33.3%) | 0.82 |
| Statin, n (%) | 59(98.3%) | 73(93.6%) | 0.23 |

Continuous variables are presented as mean ± SD or medians (25th-75th percentiles), and categorical variables are reported as counts (%). ACEIs/ARBs indicates angiotensin-converting enzyme inhibitors/angiotensin receptor blockers; BMI, body mass index; cTnI, cardiac troponin I; Hs-CRP, high-sensitivity C reactive protein; LDL-C, low-density lipoprotein cholesterol; LVEF, left ventricular ejection fraction; LVEDD, left ventricular end-diastole diameter; LVSD, left ventricular systolic dysfunction; MVD, multi-vessel disease; NT-proBNP, N-terminal pro B-type Natriuretic Peptide; PAD, peripheral artery disease; TMAO, trimethylamine-N-oxide. *Estimated glomerular filtration rate (eGFR) was calculated according to the Modification of Diet in Renal Disease formula.
